# Dynamic Oxygen Conditions Promote the Translocation of HIF-1*α* to the Nucleus in Mouse Blastocysts

**DOI:** 10.1155/2021/5050527

**Published:** 2021-09-24

**Authors:** Jungwon Choi, Wontae Kim, Hyejin Yoon, Jaewang Lee, Jin Hyun Jun

**Affiliations:** ^1^Department of Senior Healthcare BK21 Plus Program, Graduate School, Eulji University, Seongnam, Republic of Korea; ^2^Department of Biomedical Laboratory Science, Eulji University, Seongnam, Republic of Korea; ^3^Department of Biomedical Laboratory Science, Graduate School, Eulji University, Seongnam, Republic of Korea

## Abstract

Oxygen tension is one of the most critical factors for mammalian embryo development and its survival. The HIF protein is an essential transcription factor that activated under hypoxic conditions. In this study, we evaluated the effect of dynamic oxygen conditions on the expression of embryonic genes and translocation of hypoxia-inducible factor-1*α* (HIF-1*α*) in cultured mouse blastocysts. Two-pronuclear (2PN) zygotes harvested from ICR mice were subjected to either high oxygen (HO; 20%), low oxygen (LO; 5%), or dynamic oxygen (DO; 5% to 2%) conditions. In the DO group, PN zygotes were cultured in 5% O_2_ from days 1 to 3 and then in 2% O_2_ till day 5 after hCG injection. On day 5, the percentage of blastocysts in the cultured embryos from each group was estimated, and the embryos were also subjected to immunocytochemical and gene expression analysis. We found that the percentage of blastocysts was similar among the experimental groups; however, the percentage of hatching blastocysts in the DO and LO groups was significantly higher than that in the HO group. The total cell number of blastocysts in the DO group was significantly higher than that of both the HO and LO groups. Further, gene expression analysis revealed that the expression of genes related to the embryonic development was significantly higher in the DO group than that in the HO and LO groups. Interestingly, *HIF-1α* mRNA expression did not significantly differ; however, HIF-1*α* protein translocation from the cytoplasm to the nucleus was significantly higher in the DO group than in the HO and LO groups. Our study suggests that dynamic oxygen concentrations increase the developmental capacity in mouse preimplantation embryos through activation of the potent transcription factor HIF-1*α*.

## 1. Introduction

Optimized *in vitro* culture conditions for human zygotes and preimplantation embryos are vital in determining embryo developmental competency and fetus quality [1]. *In vitro*, one of the major factors that influence embryo development is the concentration of oxygen. Several studies have reported that low oxygen (LO; ≤5% oxygen) concentrations enhance the embryonic developmental competence of human zygotes and preimplantation embryos *in vitro* [2–9]. In fact, most mammals have a lower oxygen concentration in the uterus than that in the oviduct; the oxygen concentration is approximately 5-7% in the oviduct and 2% in the uterus [10–12]. Currently, there are no optimized culture systems that reflect the different oxygen concentrations within the female reproductive tract. However, *in vitro* culture systems for preimplantation embryos that mimic oxygen concentrations in the *in vivo* female reproductive system have begun to emerge [13–15], despite lack of standards for the culture systems and conditions.

High oxygen (HO) concentrations in the atmosphere may induce oxidative stress, affecting embryonic development [16]. Notably, *in vivo* preimplantation embryos are never exposed to HO concentrations, which is equivalent to the 20% oxygen concentration in the atmosphere. Previously, we found that the dynamic oxygen (DO) concentrations (decreasing from 5% to 2%) had beneficial effects on pre- and peri-implantation embryo development in mice [14]. Additionally, studies have shown that oxygen concentrations alter gene expressions in bovine and mouse preimplantation embryos [10, 17–19]. However, further investigations are still needed to estimate the effect of DO concentrations on gene expression in preimplantation embryos *in vitro*.

The hypoxia-inducible factor (HIF) family of genes encodes major transcription factors that regulate cellular metabolism in mammals to aid in sustenance and survival under hypoxic conditions [20], and the HIF family is also essential in embryonic development [21]. There are three isotypes of HIFs, HIF-1, HIF-2, and HIF-3, which are heterodimers of their *α* subunits and *β* subunits. HIF-1 activity is dependent on the stability of the *α* subunit, and HIF-1*α* is the master regulator of hypoxia-inducible genes [22–24]. During hypoxia, the HIF-1*α* cascade involving prolyl hydroxylases cannot be initiated, leading to the accumulation of HIF-1*α* protein in the cytosol. The HIF-1*α* and HIF-1*β* complex binds to the hypoxia-response element of the gene promoter then translocated to the nucleus and activated transcription [25]. At the level of the mRNA, it is difficult to observe transcription activity difference under low oxygen concentration not only in HIF-1*α* but also in HIF-2*α* protein translocation and indistinguishable compared to that under atmospheric concentrations of oxygen [26, 27]. Consequently, we proposed to observe the translocation of HIF-1*α* protein under different oxygen conditions with confocal microscopy and analyzed several developmental genes and hypoxia-affected genes in the mRNA level.

Even with the currently known advantages of LO and DO conditions in embryo culture, there is still a lack of studies that have reported the mechanism underlying the improved development of embryos under LO concentrations. Therefore, we replicated the oxygen concentrations within the female reproductive tract *in vitro*; zygotes were cultured under 5% O_2_ from the zygote to the morula stage and then shifted to 2% O_2_ to blastocyst stage on day 5 after hCG injection. We aimed to evaluate the *in vitro* effects of DO concentrations on the mRNA expression of embryonic genes and the translocation of HIF-1*α* in mouse blastocysts.

## 2. Materials and Methods

### 2.1. Animal Care and Hormonal Stimulation

Our study was approved by the ethics committee at the Eulji University Institutional Animal Care and Use Committee (EUIACUC 19-19, EUIACUC 20-12). The protocol for superovulation was applied according to our previous report [14]. Briefly, female ICR mice (aged 5-10 weeks) and male ICR mice (aged 10 weeks-3 months) were mated under controlled light-dark cycle (lights on at 8:00 AM; lights off at 8:00 PM). Before mating, female mice were superovulated with an intraperitoneal injection of 5 IU pregnant mare serum gonadotropin (Prospec, Israel) and 5 IU human chorionic gonadotropin (hCG; Prospec, Israel) 47-48 h later.

### 2.2. Collection of Two-Pronuclear (2PN) Zygotes for *In Vitro* Culture

In the morning following mating, the presence of a vaginal plug was used to confirm the event of successful mating. The primed female mice were sacrificed by cervical dislocation 19 h after hCG injection. Cumulus cells were completely denuded with a treatment of 75 U/mL hyaluronidase (Sigma-Aldrich, USA) to confirm fertilization. 2PN zygotes were washed at least 5 times with 0.4% bovine serum albumin (BSA; Sigma-Aldrich) containing a drop of KSOM (Sigma-Aldrich). We randomized retrieved embryos from one female mouse into every 3 groups of oxygen concentrations at the collection point. Five to seven embryos per 10 *μ*L media and a drop of mineral oil (Irvine, USA) were group cultured for 4 days under different oxygen conditions. Zygotes in the LO and HO groups were cultured under 5% and 20% O_2_, respectively, for 4 days. In the DO group, zygotes were cultured under 5% O_2_ conditions to the morula stage for 2 days and then cultured under 2% O_2_ to the blastocyst stage for 2 days. Nitrogen and carbon dioxide concentrations were fine-tuned using a double-channel gas supply system, also used in our previous study [14].

### 2.3. Assessment of Preimplantation Development and Immunocytochemistry of Oct-4

Preimplantation development, from the mouse 2PN zygote to the blastocyst stage, was evaluated on day 5 after hCG injection [28]. Developed blastocysts were fixed in 4% paraformaldehyde with 1% polyvinyl alcohol for immunocytochemistry (ICC). The embryos were blocked in 3% BSA (Biosesang, Seongnam, Korea) and incubated with a monoclonal mouse antibody against Oct-4 (Abcam, UK), which is expressed in the inner cell mass (ICM), overnight at 4°C. In ICC of the negative control group, the embryos were incubated without a specific primary antibody against Oct-4. On the next day, blastocysts were conjugated with Alexa 488 anti-rabbit secondary antibody (Life Technology, Norway), and the nuclei were counter stained with 4′,6-diamidino-2-phenylindole (Vector Laboratories, USA). Images were obtained using a fluorescence microscope (AX-70, Olympus, Tokyo, Japan) with IMT i-Solution program (I-solution, British Columbia, Canada). Total blastomere cell number and ICM number were counted with captured fluorescence image, and then, trophectoderm (TE) number was calculated by subtracting the number of ICM from the total cell number [29].

### 2.4. Confocal Microscopy and Quantification of HIF-1*α* Translocation in Blastocysts

The localization of HIF-1*α* protein was examined using the aforementioned protocol for immunocytochemical analysis of HIF-1*α* with an anti-HIF-1*α* primary antibody (Abcam) and an Alexa 488 anti-mouse secondary antibody (Life Technology, Norway). In ICC of the negative control group, the embryos were incubated without a specific primary antibody against HIF-1*α*. The images were obtained using a confocal microscope (Zeiss LSM 800, Oberkochen, Germany) and the ZEN software (Zeiss, Germany) with ×40 water-immersion objectives. Quantification of HIF-1*α* translocation from the cytoplasm to the nucleus was performed using ImageJ (National Institutes of Health, USA) [30]. In detail, by subtracting the DAPI image from the HIF-1*α* image using the image calculator of ImageJ program, an image of the HIF-1*α* protein present only in the cytoplasm can be obtained. Next, through the measure analysis, the nuclear/total HIF-1*α* distribution ratio can be obtained after measuring intensity density for the entire HIF-1*α* distribution image and the intensity density for the subtracted image. Note that immunofluorescence intensity density of both two images must be measured in the same area to clearly measure the subtracted area.

### 2.5. Quantitative Analysis of mRNA Expressions

Total mRNA was extracted from five blastocysts of each experimental group using the Dynabeads mRNA Direct Kit (Invitrogen, USA) according to the manufacturer's instruction. In brief, blastocysts were lysed by lysis/binding buffer and mixed with Oligo(dT)_25_ beads. This bead/mRNA complex was washed twice each through washing solutions A and B. Lastly, mRNA was separated from the complex with 20 *μ*L of 10 mM Tris-HCl and incubated at 65°C for 2 min. The cDNAs were synthesized from the whole extracted mRNA using the PrimeScript 1^st^ strand cDNA Synthesis kit (Takara Bio, Japan), and quantitative real-time polymerase chain reaction (qRT-PCR) was performed using specific primer prepared with SensiFAST SYBR Hi-ROX kit (Meridian Bioscience, USA), sequences of which are given in Table 1. The primers used in this study were optimized for 60°C annealing. Samples were run in triplicates and repeated three times. Semiquantitative analysis of mRNA expression was conducted to exclude the embryo variation and analyzed by using a modification of the 2^-*ΔΔ*ct^ method [31, 32] with internal control [33]. Each qRT-PCR tube contained 0.25 equivalent of blastocyst for target genes and 0.125 equivalent of blastocyst for internal control, respectively. All results were normalized to a reference gene, 18s ribosomal RNA, regarding embryo culture condition and blastocyst sample [34]. Expression level of each target gene was presented as fold change compared to the HO group.

### 2.6. Statistical Analysis

Differences in ratios between groups were analyzed using the chi-square test. Differences in data between groups were statistically analyzed using the one-way analysis of variance with Tukey's multiple comparison as the post hoc test. A *p* value of less than 0.05 was considered statistically significant.

## 3. Results

### 3.1. Effects of Different Oxygen Concentrations on the Development and Cell Number of Cultured Blastocysts

**Figure 1:**
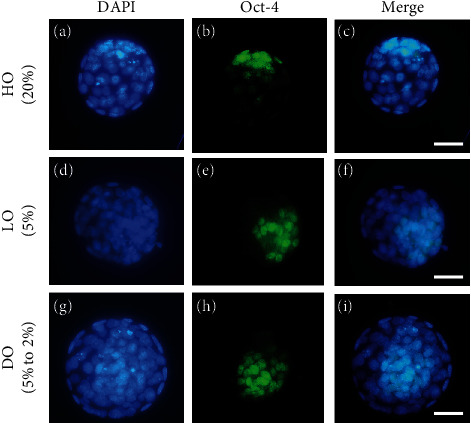
Immunocytochemistry for Oct-4 and DAPI staining for nuclei in blastocysts of *in vitro* culture with HO (20%), LO (5%), and DO (5% to 2%) conditions. Representative fluorescent microscopy images of nucleus with (a, d, g) DAPI (blue), (b, e, h) FITC-labeled Oct-4 (green), and (c, f, i) merged images. Scale bars indicate 50 *μ*m.

Mouse 2PN zygotes were cultured for 4 days, and the effect of different oxygen concentrations on the development of blastocysts was evaluated on day 5 after hCG administration (Table 2). There was no significant difference in the percentage of blastocysts on day 5 among the different experimental groups. However, the percentage of hatching blastocysts was significantly increased in the DO group (49.7%) compared to that in both the HO (32.3%) and LO (39.7%) groups (*p* < 0.01). Total cell number of blastocysts was also significantly higher in the DO group (83.6 ± 3.8) and in the LO group (75.4 ± 4.3) than that in the HO (62.4 ± 2.4) group (*p* < 0.05). The ratio of the number of cells in the ICM to the total cell number was not significantly different among the experimental groups. However, the number of cells in the ICM was significantly higher in the DO group than in the HO group (19.5 ± 1.1*vs.*14.6 ± 0.7, *p* < 0.05, Table 2).  Figure 1 shows blastomeres and inner cell mass imaged using an epi-mounted immunofluorescence microscope.

### 3.2. Effects of Different Oxygen Concentrations on Gene Expression of Cultured Blastocysts

**Figure 2:**
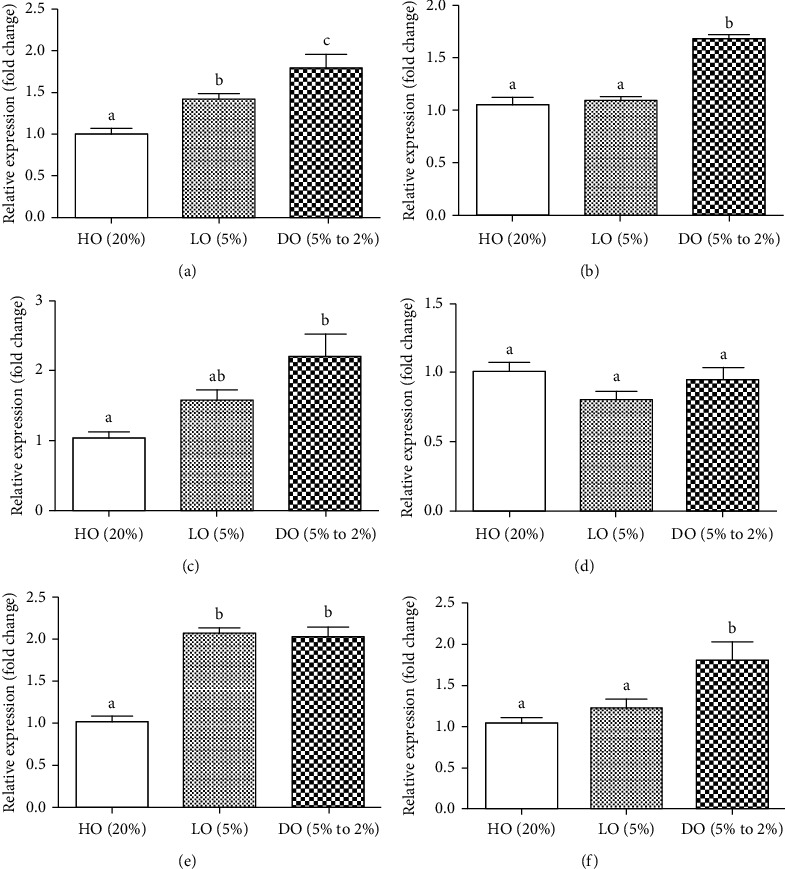
The mRNA expressions of embryonic development- and oxygen-related genes in blastocysts of *in vitro* culture with HO (20%), LO (5%), and DO (5% to 2%) conditions. The qRT-PCR analysis of (a) caudal-type homeobox 2 (Cdx-2), (b) octamer-binding transcription factor 4 (Oct-4), (c) H2A.Z variant histone 1 (H2az1), (d) hypoxia-inducible factor-1*α* (HIF-1*α*), (e) manganese superoxide dismutase (MnSOD), and (f) 16s ribosomal RNA (16s rRNA) in blastocysts of *in vitro* culture with HO, LO, and DO conditions. Values are represented as mean ± SEM for each group and analyzed by one-way ANOVA using Tukey's multiple comparison test. Different letters (a, b, c) indicate statistically significant differences (*p* < 0.05).

The expressions of differentiation-related genes in blastocysts, such as *Cdx2* and *Oct-4*, were significantly higher in the DO group than in the HO and LO groups. The expression level of the histone gene *H2az1* was significantly increased in the DO group compared to the HO group. The expression level of *HIF-1α* was not significantly different between the three experimental groups. However, the expression level of reactive oxygen species- and mitochondrial activity-associated genes, *MnSOD* and *16*s *rRNA*, was significantly increased in the DO group compared with the HO group. Interestingly, the expression level of *MnSOD* was also significantly higher in the LO group than in the HO group (Figure 2).

### 3.3. Effects of Different Oxygen Concentrations on HIF-1*α* Translocation Ratio in *In Vitro* Cultured Blastocysts

**Figure 3:**
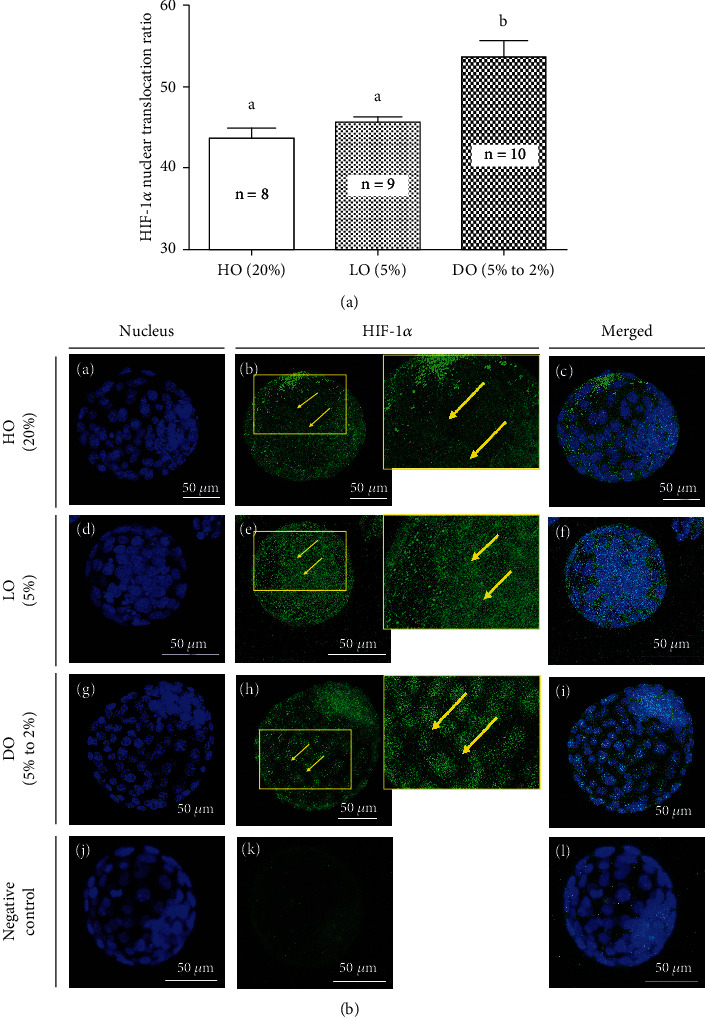
Translocation of HIF-1*α* from the cytoplasm into the nucleus in mouse blastocysts of *in vitro* culture with HO (20%), LO (5%), and DO (5% to 2%) conditions. (a) Quantitative analysis of HIF-1*α* translocation ratio in epi-mounted immunofluorescence images by nuclear to total cell intensity. Values are represented as mean ± SEM for each group and analyzed by one-way ANOVA using Tukey's multiple comparison test. Different letters indicate statistically significant differences (*p* < 0.05). (b) Representative confocal microscopy images of the nucleus with DAPI (A, D, G, J), Alexa 488-labeled HIF-1*α* (B, E, H, K), and merged images (C, F, I, L). Scale bars indicated 50 *μ*m.

The HIF-1*α* protein localization of *in vitro* blastocysts cultured under different oxygen concentrations was visualized and quantified using epi-mounted immunofluorescence (Figure 3(a)). The translocation ratio was significantly different between that of the DO group (53.7%, *n* = 10) and that of the LO (45.7%, *n* = 9) and HO (43.7%, *n* = 8) groups. This difference was more obvious when translocation was visualized and quantified using confocal microscopy. HIF-1*α* was translocated to the nucleus from the cytoplasm in both the LO and DO groups. We found that the translocation of HIF-1*α* protein was significantly increased under DO concentrations compared to under LO concentrations, even though HIF-1*α* mRNA levels were comparable between both groups.

## 4. Discussion

Many studies suggested that LO (under 5% O_2_) conditions are beneficial for preimplantation embryo development in various mammals, including mouse and human [2–9]. However, in this study, we showed that the percentage of blastocysts was not significantly different among the HO, LO, and DO groups. Our results were similar to the reports of Karagenc et al., wherein they also showed that there was no significant difference in blastocyst development between 20% O_2_ and 5% O_2_ in mouse PN zygotes [35]. Additionally, Feil et al. investigated the effect of alteration of oxygen concentration (DO conditions) from 7% O_2_ for 3 days to 20%, 7%, or 2% O_2_ for 2 days on development of mouse embryos and found that there were no significant differences among the three groups [13]. A gradual shift in oxygen concentration from 5% to 2% O_2_ on day 3, like our DO group, had no significant difference in the percentage of blastocyst formation of human ICSI embryos compared embryos under 5% O_2_ [4]. Despite an absence of difference in the percentage of blastocyst formation and hatching blastocysts on day 5, it was significantly increased in the DO group compared to the HO and LO groups, suggesting that DO concentrations could improve the peri-implantation embryo development competence [36].

We found an increase of total cell number of blastocysts in the LO and DO groups compared to the HO group (Table 2). This increase in cell number under LO (5% O_2_) concentrations has been reported by several studies [8, 35, 37]. We speculated that the increase in cell number under LO and DO conditions could be related to cell cycle progression [38, 39]. Wale and Gardner reported that a shift in 20% to 5% oxygen concentrations did not show similar results in the percentage of blastocysts and cell number when compared to that of continuous culture under 20% oxygen. They suggested that hyperoxygen condition affects up to the 8-cell stage in mouse embryos [37].

In this study, we analyzed six genes for two main categories by semiquantitative RT-PCR using internal control. We selected the housekeeping gene by considering several factors such as type of mouse, type of culture medium, and the purpose of the study [34]. There were GAPDH and *β*-actin as internal control gene candidates, but the former had a pseudogene and the latter was actually greatly affected by genomic DNA contamination [40]. For this reason, we selected 18s rRNA as internal control in this study. We confirmed that the results of Table 2 were consistent with mRNA expression patterns of *Cdx2* and *Oct-4* through qRT-PCR. Several mRNAs related to the embryonic development quantified by qRT-PCR were significantly increased in blastocysts cultured under DO conditions (Figure 2). Expression level of *Cdx2*, which is an important transcription factor in the trophectoderm, was increased in the DO (*p* < 0.001) and LO (*p* < 0.05) groups compared with the HO group. Additionally, gene expression of *Oct-4* in the ICM showed significant increase in the DO group compared with the LO and HO groups [41]. These results are consistent with the results of Table 2 and Figure 1 that show an increase in the number of cells in the ICM. Next, we analyzed *H2az1* gene, which plays an important role in early embryonic development and is widely distributed in molecules depending on oxygen conditions like hypoxia. *H2az1* is one of the H2A.Z variants of histone 1 that is related to nuclear protein and transcription activity during the preimplantation period [42]. *H2az1* plays a critical role in early embryo development since it is involved in establishing chromatin structure [43]. In particular, Dong et al. found that H2A.Z was enriched in genes related to signaling pathway of response to oxygen level [44]. Our result also showed the mRNA expression of *H2az1* in the DO group was significantly higher than that in the HO group. We also analyzed the expression of genes associated with oxygen metabolism such as *MnSOD* and *16*s *rRNA*. *MnSOD* is an important mitochondrial antioxidant gene, and *16s rRNA* is the ribosomal RNA which regulates mitochondrial translation [45, 46]. Hypoxia condition activates HIF-1*α* through stabilization of the HIF*α* subunit, and this leads to increasing of *FOXO* gene activity [47]. In particular, the expression of *FOXO3a* and its downstream genes is also increased, such as MMP family, antioxidant, and cell programing genes [48]. Among these genes, we analyzed the antioxidant-related gene, *MnSOD*. Interestingly, *FOXO3a* could be activated by ROS, but also by HIF-1*α* stabilization [49–51]. However, most hypoxia research subjects are cancer cell, and it could have different biological mechanism from that of normal cell. Several studies investigated that increase of MnSOD in *C. elegans* and murine expression was linked to increased lifespan [49, 52–54]. Blastocysts cultured in the LO and DO groups had higher levels of *MnSOD* mRNA than those in the HO group. This result is consistent with that of the study in bovine and mouse [26, 55]. The DO and LO conditions lead to enhanced antioxidant defense against oxidative stress during development processes than HO conditions [26]. Mitochondria are the most important organelle in the embryo, and there is a strong correlation between mitochondrial function and the development of the human embryo [56, 57]. Synthesis of ATP and oxidation of glucose by respiration need more energy and produce more ROS than glycolysis. There is a study that in mouse embryo fibroblasts (MEFs) exposed to hypoxia for several days, ROS level was decreased compared to 20% oxygen culture condition. However, HIF-1a knockout MEFs under chronic hypoxia causes ROS level rather than decrease [58]. Cellular RNA is more susceptible to these ROS than DNA, and this damage can affect RNA structure or RNA function. Therefore, if the antioxidant mechanism is not carried out smoothly, increased ROS could lead to RNA damage [59]. Further, several studies have demonstrated that HO concentrations induce imbalance between ROS generation and ROS scavenging in live cells [55, 60, 61]. In our study, blastocysts cultured under DO exhibit significantly higher mRNA levels of *16*s *rRNA* compared with that in the HO group. Comprehensively, DO concentrations are favorable for the survival and development of preimplantation embryos.

The HIF protein is an essential transcription factor activated under hypoxic conditions. Among the HIF subtypes, HIF-1*α* is the master transcription factor that promotes cell survival, proliferation, angiogenesis, and metabolism [62]. Accordingly, there are studies that have reported that a *HIF-1a* deficiency can result in embryo lethality [63–65]. In this study, there was no significant difference in mRNA expression of *HIF-1α* among the different experimental groups. Since HIF-1*α* protein accumulates in the cytosol and translocated to the nucleus under hypoxic conditions and induces transcription of downstream genes [25], we evaluated HIF-1*α* protein distribution in blastocysts cultured under the three different oxygen conditions. We found that the translocation ratio was significantly increased in the DO group compared with both the LO and HO groups. Furthermore, this difference was more obvious upon confocal microscopic analysis, as shown in Figure 3(b). HIF-1*α* distribution in both blastocysts of both the DO and LO groups has a unique pattern such that its translocation is indistinguishable in the area around the ICM. Since the ICM is located inside the blastocyst and has a higher cell density than the trophoblast, we hypothesize that the area around the ICM is less susceptible to changes in oxygen concentrations.

A limitation of this study is that effect of exposure to 20% oxygen concentrations could not be ruled out during handling and sampling of the 2PN stage zygote embryos. Future studies are needed to optimize the oxygen condition for appropriate HIF-1*α* activity of preimplantation embryos, especially in human *in vitro* fertilization-embryo transfer programs. Additionally, the influence of dynamic oxygen condition should be further investigated in fetal and placental development after implantation.

## 5. Conclusion

Collectively, this study demonstrated that dynamic oxygen concentration of 5% for 2 days, from PN to compaction stage, and 2% for 2 days, from compaction to blastocyst stage, could promote the development of mouse preimplantation embryos. In particular, DO (5% to 2%) conditions *in vitro* showed an increase in the percentage of hatching blastocysts, mRNA expressions of embryonic development-related genes, and nuclear translocation of HIF-1*α* than in the HO (20%) and LO (5%) conditions. Therefore, we suggest that the *in vitro* oxygen concentrations for preimplantation development should be precisely optimized to enhance the *in vitro* cultured embryo quality in human *in vitro* fertilization-embryo transfer programs.

## Figures and Tables

**Table 1:** Primer lists for qRT-PCR used in this study.

Genes	Primer sequences	Product size	GenBank accession number
*Cdx2* (*caudal type homeobox 2*)	Forward: 5′-GCAGTCCCTAGGAAGCCAAGTGA-3′	162 bp	NM_007673.3
	Reverse: 5′-CTCTCGGAGAGCCCAAGTGTG-3′		
*Oct-4* (*octamer-binding transcription factor 4*)	Forward: 5′-TCAGGTTGGACTGGGCCTAG-3′	100 bp	NM_013633.3
	Reverse: 5′-GGAGGTTCCCTCTGAGTTGC-3′		
*H2az1* (*H2A.Z variant histone 1*)	Forward: 5′-CGTATCACCCCTCGTCACTT-3′	286 bp	NM_016750.3
	Reverse: 5′-AAGCCTCCAACTTGCTCAAA-3′		
*HIF-1α* (hypoxia-inducible factor-1*α*)	Forward: 5′-CTTCTGGATGCCGGTGGTCTAGAC-3′	254 bp	NM_001313919.1
	Reverse: 5′-CTCACTGGGCCATTTCTGTGTGTAAG-3′		
*MnSOD* (manganese superoxide dismutase)	Forward: 5′-GCACATTAACGCGCAGATCA-3′	241 bp	NM_013671.3
	Reverse: 5′-AGCCTCCAGCAACTCTCCTT-3′		
*16s rRNA* (16s ribosomal RNA)	Forward: 5′-AGATGATCGAGCCGCGC-3′	163 bp	NM_013647.2
	Reverse: 5′-GCTACCAGGGCCTTTGAGATGGA-3′		
*18*s *rRNA* (18s ribosomal RNA)	Forward: 5′-GCCCTGTAATTGGAATGAGTCCACTT-3′	149 bp	NR_003278.3
	Reverse: 5′-GCTCCCAAGATCCAACTACGAGCTTT-3′		

**Table 2:** Effects of different oxygen concentrations on the development of preimplantation embryos and cell number of blastocysts.

On day 5 after hCG injection	HO (20%)	LO (5%)	DO (5% to 2% O_2_)
Percentage of blastocysts	70.5%^a^ (124/176)	73.9%^a^ (136/184)	72.7%^a^ (149/205)
Percentage of hatching blastocysts	32.3%^a^ (40/124)	39.7%^a^ (54/136)	49.7%^b^ (74/149)
Number of cells in ICM^†^	14.6 ± 0.7^a^ (*n* = 28)	17.4 ± 1.2^ab^ (*n* = 33)	19.5 ± 1.1^b^ (*n* = 30)
Number of cells in TE^†^	47.8 ± 2.5^a^ (*n* = 28)	58.0 ± 3.8^ab^ (*n* = 33)	64.1 ± 3.4^b^ (*n* = 30)
Total cell number^†^	62.4 ± 2.4^a^ (*n* = 28)	75.4 ± 4.2^a^ (*n* = 33)	83.6 ± 3.8^b^ (*n* = 30)
ICM/total cell number ratio^†^	24.2 ± 1.43^a^ (*n* = 28)	24.0 ± 1.3^a^ (*n* = 33)	24.1 ± 1.3^a^ (*n* = 30)

A total of 49 female mice were corresponded to the blastocysts. Percentages for blastocyst formation and hatching were analyzed by SPSS statistics. ^†^Values are represented as mean ± SEM for each group and analyzed by one-way ANOVA using Tukey's multiple comparison test. HO: high oxygen (20%); LO: low oxygen (5%); DO: dynamic oxygen (5% to 2%); ICM: inner cell mass; TE: trophectoderm. ^a,b^Different superscript letters indicate statistically significant differences (*p* < 0.05).

## Data Availability

The authors confirm that the data supporting the findings of this study are available within the article.
